# CD74 Correlated With Malignancies and Immune Microenvironment in Gliomas

**DOI:** 10.3389/fmolb.2021.706949

**Published:** 2021-09-01

**Authors:** Shengchao Xu, Xizhe Li, Lu Tang, Zhixiong Liu, Kui Yang, Quan Cheng

**Affiliations:** ^1^ Department of Neurosurgery, Xiangya Hospital of Central South University, Changsha, China; ^2^ Department of Thoracic Surgery, Xiangya Hospital of Central South University, Changsha, China; ^3^ Department of Clinical Pharmacology, Xiangya Hospital, Central South University, Changsha, China; ^4^ National Clinical Research Center for Geriatric Disorders, Xiangya Hospital, Central South University, Hunan, China

**Keywords:** CD74, glioma, prognosis, diagnosis, immune microenvironment

## Abstract

**Background:** Cluster of differentiation 74 (CD74) is found to be highly involved in the development of various types of cancers and could affect the activities of infiltrated cells in the tumor microenvironment. However, these studies only focus on a few types of immune cells. Our study aims to comprehensively explore the role of CD74 in glioma prognosis and immune microenvironment.

**Methods:** A total of 40 glioma specimens were collected in this study. We extracted data from The Cancer Genome Atlas (TCGA), Chinese Glioma Genome Atlas (CGGA), and Gene-Expression Omnibus (GEO) databases to explore the expression pattern of CD74 in gliomas. gene sets enrichment analysis and gene set variation analysis analyses were conducted to characterize the immune features of CD74. ESTIMATE, ssGSEA, Tumor IMmune Estimation Resource, and CIBERSORT algorithms were applied to assess the immune infiltration. Kaplan-Meier analysis was used for survival analysis. Receiver operating characteristic analysis was used to evaluate the predictive accuracy of CD74 in glioma diagnosis and prognosis.

**Results:** A total of 2,399 glioma patients were included in our study. CD74 was highly expressed in glioma tissue compared to normal brain tissue and its expression was significantly higher in the high-grade glioma compared to the lower grade glioma at transcriptional and translational levels. Besides, CD74 was positively associated with immune checkpoints and inflammatory cytokines as well as immune processes including cytokine secretion and leukocyte activation. The high expression of CD74 indicated a high infiltration of immune cells such as macrophages, dendritic cells, and neutrophils. Moreover, patients with high expression of CD74 had poor prognoses. CD74 had moderate predictive accuracy in the diagnosis of glioblastoma and prediction of survival.

**Conclusions:** In conclusion, our study revealed that the high expression of CD74 was associated with poor prognosis and high immune infiltration. CD74 could be used as a potential target for glioma treatment and as a biomarker to predict the prognosis of glioma patients.

## Introduction

Gliomas are primary tumors derived from the glial cells in the central nervous system, which comprise about 80% of malignant brain tumors (Goodenberger and Jenkins, 2012). According to the World Health Organization classification, gliomas are categorized into four grades: grade I, II, III, and IV (Louis et al., 2016). Although great progress has been achieved to develop novel strategies for the treatment of cancers, the prognosis of glioma patients remains unsatisfactory. The median survival time of patients with grade II, III gliomas was 11.6 and 3 years, respectively, whereas that grade IV glioma is about 15 months (Ohgaki and Kleihues, 2005; Bleeker et al., 2012; Smoll et al., 2012; Thakkar et al., 2014). Therefore, there is clear urgent to develop novel approaches for the treatment of gliomas.

Several biomarkers have been found to predict the prognoses of glioma patients with potent value. Previous studies revealed that patients with isocitrate dehydrogenase (IDH) mutant or 1p19q codeletion glioma had relatively favorable prognoses (Molenaar et al., 2014a; Molenaar et al., 2014b; Chen et al., 2019). Moreover, patients with methylated O6-methylguanine-DNA methyltransferase (MGMT) promoter gliomas benefit much more from temozolomide and radiotherapy (Hegi et al., 2005). Since the prognosis of glioma patients remains poor, more biomarkers are needed to predict the survival of glioma patients.

The cluster of differentiation 74 (CD74) is a polypeptide as the invariant chain of human lymphocyte antigen (HLA) class II. It is highly involved in the antigen presentation and the activation of CD4^+^ T cells (Cresswell, 1994). CD74 is the cell surface membrane receptor for the cytokine macrophage migration inhibitory factor (MIF) (Farr et al., 2020). The role of the interaction between CD74 and MIF has been revealed in different types of cancers (Binsky et al., 2007; Nagata et al., 2009). The inhibition of CD74 and MIF could significantly attenuate the tumor growth of prostate cancer cells and melanoma mice model (Meyer-Siegler et al., 2006; Tanese et al., 2015). In the tumor microenvironment, the inhibition of the CD74-MIF signaling pathway could restore the antitumor activity of macrophage and dendritic cells against melanoma (Figueiredo et al., 2018).

Previous studies indicated that CD74 was highly expressed in high-grade gliomas, and it was associated with the microenvironment of glioma and could facilitate the proliferation of glioma cells (Zeiner et al., 2015; Ghoochani et al., 2016; Alban et al., 2020). However, these studies only explore the association between CD74 and different subtypes of macrophages, whereas no study has comprehensively characterized the prognostic and immune features in gliomas. Therefore, our study extracted 2,399 glioma samples from The Cancer Genome Atlas (TCGA), Chinese Glioma Genome Atlas (CGGA), and Gene-Expression Omnibus (GEO) databases to characterize the association between CD74 and glioma prognosis as well as immune microenvironment via the application of various algorithms. Through the analysis based on a large number of samples, our study would provide a novel insight into the immune characteristics of CD74 in gliomas and reveal a potential therapeutic target as well as a biomarker for gliomas.

## Methods

### Data Extraction

RNA expression of 2,399 glioma patients with corresponding clinical data was extracted from TCGA (https://portal.gdc.cancer.gov/), CGGA (http://www.cgga.org.cn/), and GEO (https://www.ncbi.nlm.nih.gov/geo/) databases. The TCGA-LGG, TCGA-GBM, CGGA325, CGGA693, CGGA301, and GSE108474 datasets were included in our study. TCGA-LGG and TCGA-GBM datasets were combined and defined as the TCGA dataset, and CGGA325 and CGGA693 datasets were combined and defined as the CGGA dataset for further analysis. There were 672, 1,013, 300, and 414 samples in the TCGA, CGGA, CGGA301, and GSE108474 datasets, respectively (Table 1). The batch effect was evaluated and addressed using “SVA” R package. RNA expression of normal brain tissue was obtained from Genotype-Tissue Expression (GTEx) database, which was analyzed using GEPIA online database (Tang et al., 2017). The TCGA and CGGA were RNA-seq datasets whereas CGGA301 and GSE108474 were microarray datasets. The TCGA (FPKM) dataset was downloaded format using “TCGAbiolinks” R package. CGGA (FPKM) and CGGA301 datasets were obtained from the official website. The molecular subtype of gliomas was downloaded from TCGA and CGGA databases based on definition proposed by Verhaak et al. (2010). All expression values were transformed into log_2_ (transcripts per kilobase million (TPM)+1). The characteristics of glioma patients included in our study were summarized in Table 1. The gene list of immune checkpoints and inflammatory cytokines was selected from previous studies (Wang et al., 2019; Xu et al., 2020a).

**TABLE 1:** Characteristics of patients included in our study.

	TCGA (*n* = 672)	CGGA (*n* = 1,013)	CGGA301 (*n* = 300)	GSE108474 (*n* = 414)
Age				
≤41	290	469	144	—
>41	382	543	154	—
NA	0	1	2	—
Gender				
Male	387	597	179	—
Female	285	416	121	—
Subtype				
Classical	83	—	23	—
Mesenchymal	98	—	111	—
Proneural	242	—	86	—
Neural	112	—	80	—
NA	137	—	0	—
Grade				
I	0	0	0	2
II	256	290	116	86
III	265	333	57	82
IV	150	385	124	124
NA	1	5	3	120
IDH status				
Mutant	434	528	133	—
Wildtype	228	434	165	—
NA	10	51	2	—
MGMT				
Methylated	478	469	98	—
Unmethylated	157	374	187	—
NA	37	170	15	—
1p/19q status				
Codel	171	212	16	—
Non-codel	497	728	76	—
NA	4	73	208	—

IDH: isocitrate dehydrogenase; MGMT: O-6-methylguanine-DNA methyltransferase.

### Glioma Specimens and Immunohistochemistry

A total of 40 glioma specimens including 21 grade II, 8 grade III, and 11 grade IV gliomas were collected to detect the protein level of CD74, which was revealed by immunohistochemistry (IHC). All the tumors were primary glioma and samples were collected when patients first received surgery without previous chemotherapy or radiotherapy. Our study was approved by the Ethics Committee of Xiangya Hospital, Central South University with written informed consent obtained. The specimens were embedded in paraffin sections. Citrate buffer (pH = 6.0) was applied for antigen retrieval. Then 0.3% H_2_O_2_ and 5% BSA were used for the blockade. The CD74 antibody (A9149, ABclonal, 1:100) was applied at 4 centigrade overnight. After the application of secondary antibody, diaminobenzidine tetrahydrochloride (DAB) and hematoxylin were used for staining. The score of CD74 expression was calculated by intensity score * quantity score. As for intensity scores, 0, 1, 2, and three represented negative, weak, moderate, and strong, respectively. The quantity score was determined by the proportion of stained cells, in which 0, 1, 2, 3, and four represented <10%, 10–25%, 25–50%, 50–75%, >75%, respectively. For different scores, 1–4, 5-8, and 9–12 indicated “+“, “++“, and “+++“, respectively.

### Bioinformatic Analyses

The expression of CD74 in glioma and normal tissue was compared on the GEPIA website, which contained gene expression data of TCGA and GTEx databases (Tang et al., 2017). The gene set variation analysis (GSVA) enrichment analysis was performed to show the biological processes using the “GSVA” R package (Hänzelmann et al., 2013). The co-expressed genes were screened based on the “spearman” method. Genes with |r|>0.5 and *p* < 0.001 were selected. The gene sets enrichment analysis (GSEA) enrichment analysis was conducted using “clusterProfiler” R package in Gene Ontology (GO) terms and Kyoto Encyclopedia of Genes and Genomes (KEGG) pathways. During the GSEA enrichment analysis, the value of “r” was defined as the log_2_ (fold change) and the *p*-value was defined as the false discovery rate (FDR). The infiltration of stromal and immune cells and tumor purity were assessed by Estimation of STromal and Immune cells in MAlignant Tumor tissues using Expression data (ESTIMATE) algorithm using “estimate” R package (Yoshihara et al., 2013). The estimation of immune cell infiltration was conducted by the single-sample gene-set enrichment analysis (ssGSEA), Tumor IMmune Estimation Resource (TIMER), and CIBERSORT algorithms, in which ssGSEA contained 28 immune cells, the TIMER contained six immune cells, and CIBERSORT contained 22 immune cells (Barbie et al., 2009; Newman et al., 2015; Li et al., 2020). A transcription factor (TF) - target gene interaction mapping analysis was conducted using ARACNE algorithm (Margolin et al., 2006).

### Statistical Analysis

All data were analyzed and visualized using R version 3.6.0 (https://www.r-project.org/) and GraphPad Prism version 8.0.1. Shapiro-Wilk test was used to assess the normality of data. The comparison of the difference between the two groups was conducted using Student’s t-test and Wilcoxon test. The one-way ANOVA test and Kruskal-Wallis test were used to compare the difference between three or more groups. Kaplan-Meier analysis was used to compare the survival time of patients in two groups. Multivariate Cox analysis was conducted to evaluate the prognostic value of CD74 in the overall survival (OS) of glioma patients in consideration of various clinical features. Receiver operating characteristic (ROC) analysis was conducted to evaluate the predictive accuracy of CD74 in the classification of glioblastoma (GBM). Time-dependent ROC analysis was adopted to assess the predictive accuracy of CD74 in the survival of glioma patients. The correlation between CD74 and other gene expressions was determined by the “spearman” method. The Two-sided *p*-value < 0.05 was considered statistically significant.

## Results

### High Expression of CD74 Was Associated With Glioma Malignancies

**FIGURE 1:**
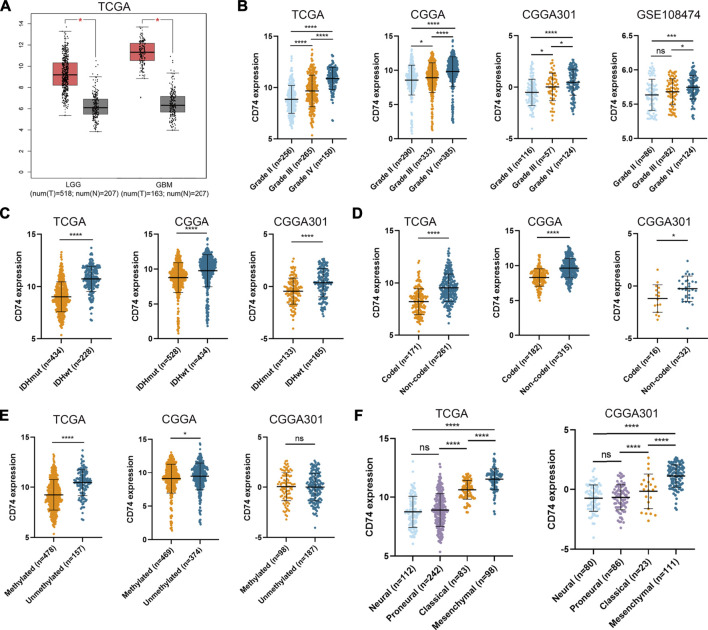
Expression of CD74 was associated with glioma malignancies. **(A)** Expression of CD74 in glioma and normal brain tissues. The red and grey box represented tumor and normal tissues, respectively. B-E. CD74 expression in different grades **(B)**, IDH status **(C)**, 1p19q status **(D)**, and MGMT status **(E)** of gliomas. F. Expression of CD74 in different molecular subtypes of glioma. G Immunohistochemistry staining of CD74 in glioma and normal brain tissues. Data were represented as mean ± standard deviation by dot plot. IDH: isocitrate dehydrogenase; MGMT: O6-methylguanine-DNA methyltransferase. *, *p* < 0.05; ***, *p* < 0.001; ****, *p* < 0.0001; ns, no significance.

**FIGURE 2:**
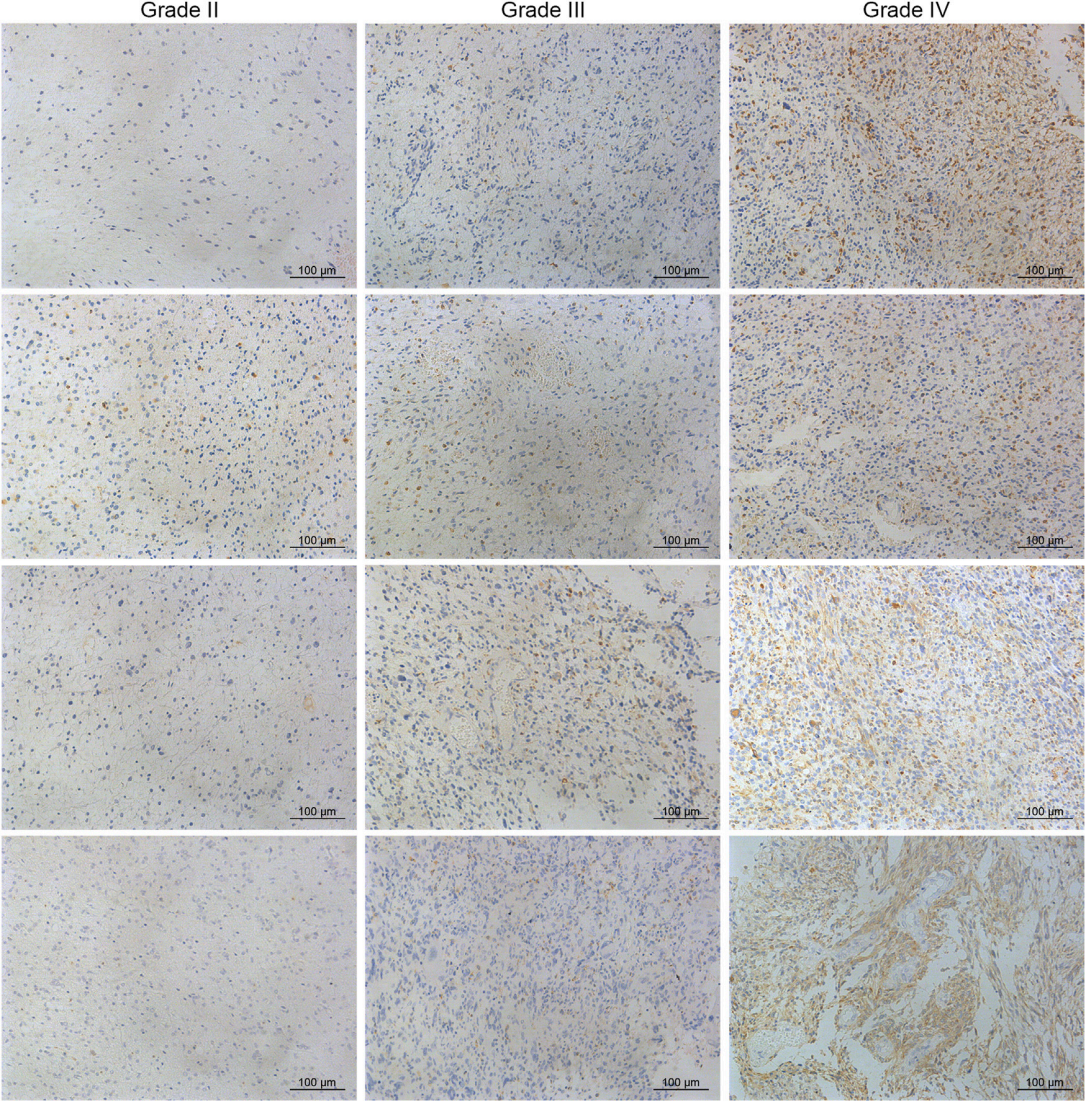
Immunohistochemistry of CD74 in different grades of gliomas.

To characterize the role of CD74 in gliomas, we explored the expression pattern of CD74 in gliomas and normal tissue. In both lower-grade glioma (LGG) and GBM, the expression of CD74 was significantly higher compared to the normal tissue (*p* < 0.05) (Figure 1A). Shapiro-Wilk test indicated that the expression of CD74 in each dataset did not all follow normal distribution. Therefore, Kruskal-Wallis test revealed that the expression of CD74 was significantly elevated in grade IV glioma compared with grade II and III gliomas in the TCGA, CGGA, CGGA301, and GSE108474 datasets (*p* < 0.05) (Figure 1B). In grade II and III gliomas, CD74 had a distinct expression pattern in TCGA, CGGA, and CGGA301 datasets but not in GSE108474 dataset (Figure 1B). In different subtypes of glioma, the expression of CD74 was significantly higher in the IDH wildtype and 1p19q non-codeletion gliomas (*p* < 0.05) (Figures 1C,D). As for MGMT methylation, CD74 was highly expressed in unmethylated gliomas than methylated gliomas in TCGA and CGGA datasets (*p* < 0.05), whereas this trend was not detected in the CGGA301 dataset (*p* > 0.05) (Figure 1E). Besides, in different molecular subtypes of gliomas, the expression of CD74 had no significant difference between neural and proneural gliomas (*p* > 0.05) but was significantly increased in the mesenchymal subtype compared with other subtypes of glioma (*p* < 0.05) (Figure 1F). Since IDH mutation, MGMT methylation, and 1p19q codeletion indicated high malignancies and poor prognosis, the expression of CD74 might be associated with glioma malignancies. Apart from the RNA level, we also investigated the expression of CD74 at the protein level in different grades of gliomas. The IHC showed that the expression of CD74 was higher in the higher grade of glioma and was highest in grade IV glioma specimens (Figure 2; Table 2). These results indicated that CD74 was associated with glioma malignancies and might be used as a potential biomarker.

**TABLE 2:** IHC score of CD74 in different grades of gliomas.

	+	++	+++	*p* Value
Grade II	14	5	2	<0.001
Grade III	1	5	2	
Grade IV	2	1	8	

### Expression of CD74 Correlated With Immune-Related Genes and Immune Processes

**FIGURE 3:**
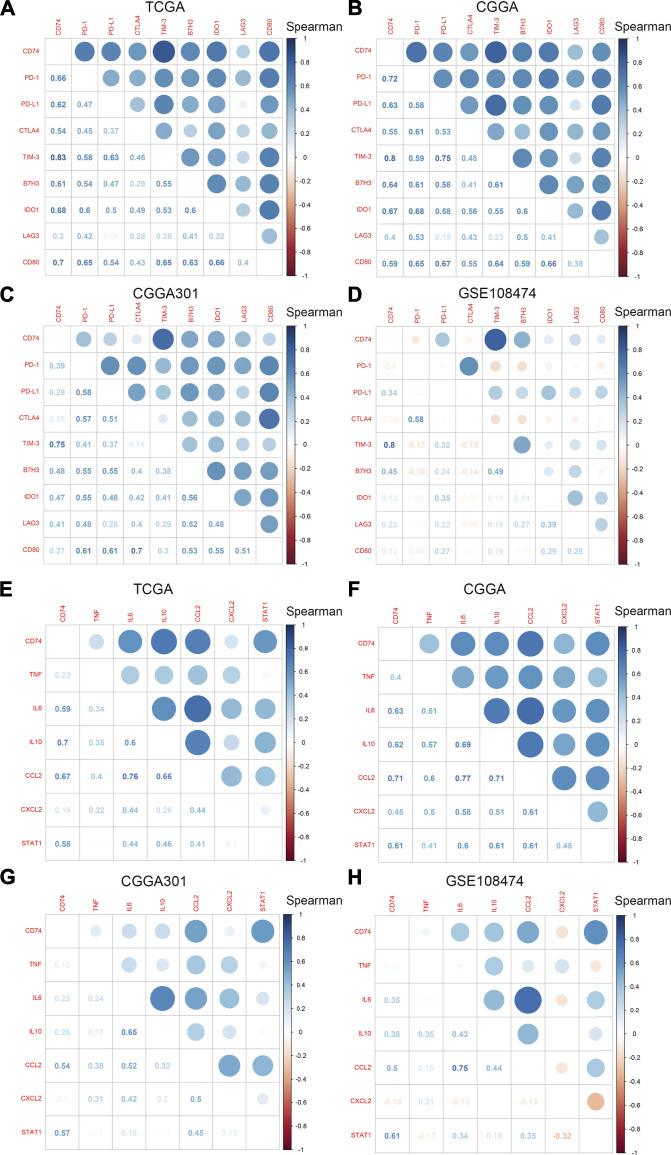
Correlation between CD74 and immune checkpoints and inflammatory cytokines. A-D. Correlation between CD74 and immune checkpoints in TCGA **(A)**, CGGA **(B)**, CGGA301 **(C)**, and GSE108474 **(D)** datasets. E-H. Correlation between CD74 and inflammatory cytokines in TCGA **(E)**, CGGA **(F)**, CGGA301 **(G)**, and GSE108474 **(H)** datasets. Blue and red represented positive and negative correlation, respectively.

**FIGURE 4:**
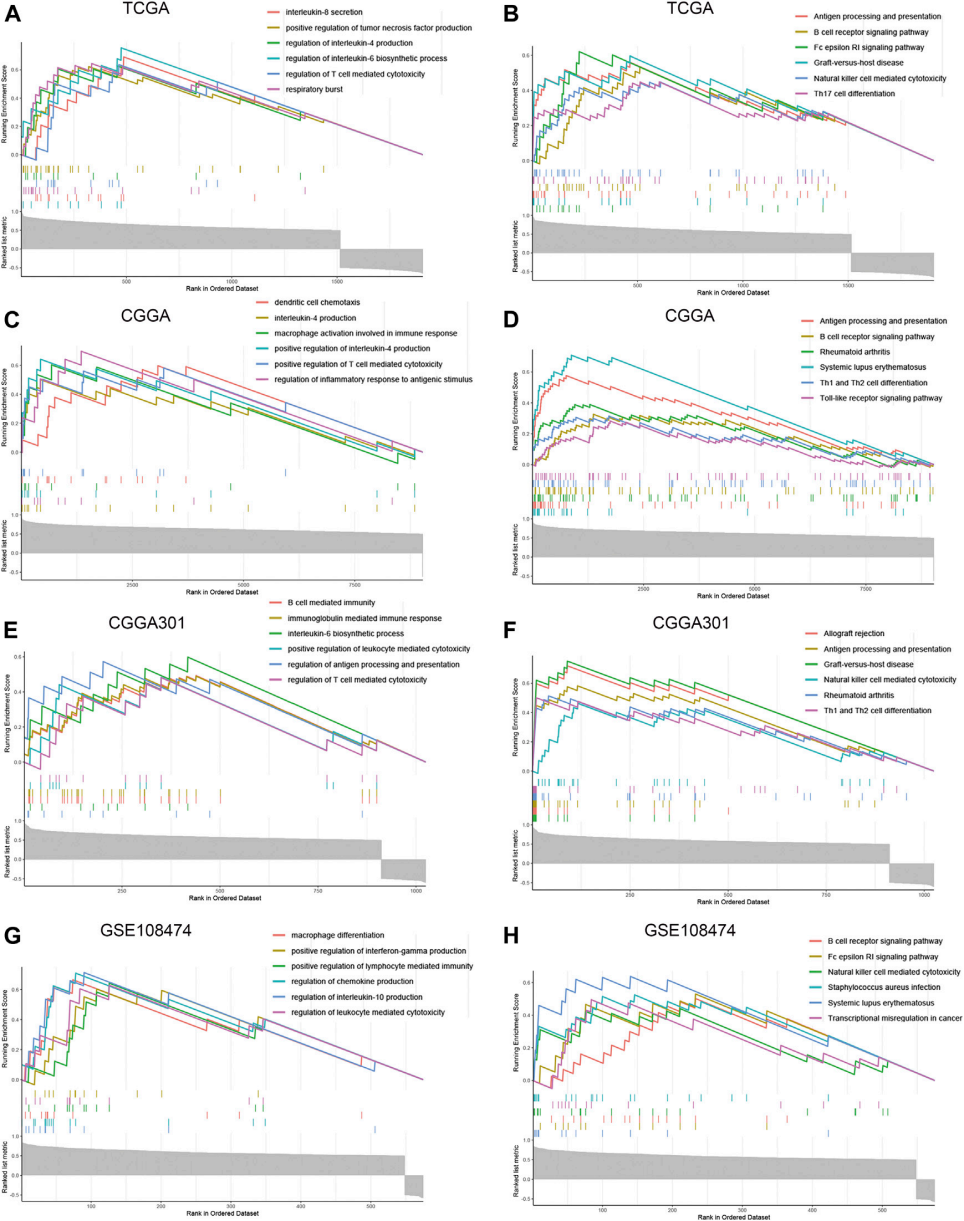
Enrichment analysis of co-expressed genes of CD74. A-H. GSEA enrichment analysis of co-expressed genes of CD74 in GO and KEGG terms in the TCGA **(A, B)**, CGGA **(C, D)**, CGGA301 **(E, F)**, and GSE108474 **(G, H)** datasets. GO: Gene Ontology; KEGG: Kyoto Encyclopedia of Genes and Genomes.

**FIGURE 5:**
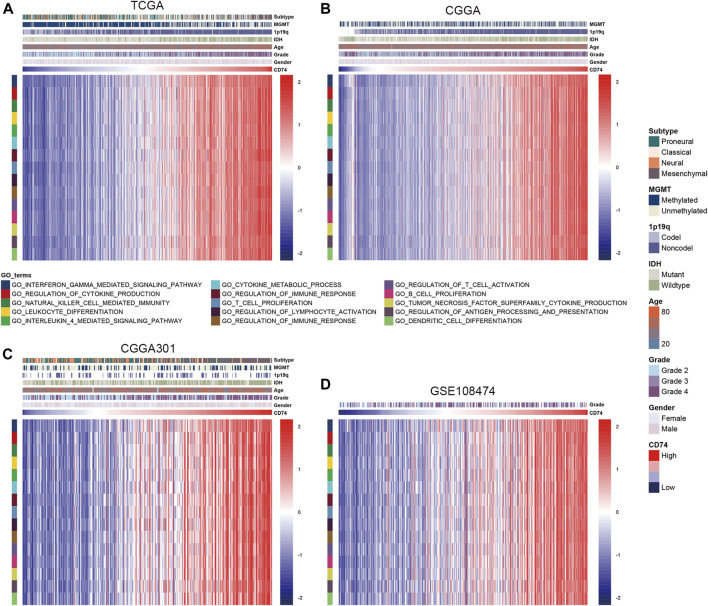
Association between CD74 expression and immune processes. A-D. GSVA analyses revealed the association between the enrichment of immune processes and CD74 expression in the TCGA **(A)**, CGGA **(B)**, CGGA301 **(C)**, and GSE108474 **(D)** datasets. Red and blue represented relatively high and low involvement in each pathway.

To further explore the characteristics of CD74, we assessed the correlation between CD74 and immune-related genes such as immune checkpoints and inflammatory cytokines. The gene list of immune checkpoints and inflammatory cytokines was selected from previous studies (Wang et al., 2019; Xu et al., 2020a). Results showed that CD74 was positively associated with the expression of immune checkpoints such as PD-1, PD-L1, IDO1, and B7H3 in TCGA, CGGA, and CGGA301 datasets (Figures 3A–C). The expression of CD74 was notably associated with the expression of T cell immunoglobulin domain and mucin domain-3 (TIM-3), which could lead to the suppression of T cells (Figure 3D) (Blackburn et al., 2009). Moreover, CD74 was positively correlated with the expression of inflammatory cytokines such as IL-6, IL-10, and CCL2 in the TCGA, CGGA, CGGA301, and GSE108474 datasets (Figures 3E–H). Then, we screened the co-expressed genes of CD74 with the cutoff point of |r|>0.5 and *p* < 0.001 to further characterize the role of CD74. Enrichment analysis revealed that the co-expressed genes were enriched in cytokine secretion, immune cell mediated cytotoxicity, and inflammatory response to antigenic stimulation in GO biological processes; as for in KEGG pathway, they were highly involved in antigen processing and presentation, Th1 and Th2 cell differentiation, natural killer cell mediated cytotoxicity, and autoimmune diseases such as rheumatoid arthritis and systemic lupus erythematosus (Figures 4A–H). Meanwhile, ARACNE algorithm was conducted to screening co-expressed genes of CD74 that were controlled by the distinct transcriptional factors, in which immune-associated genes such as IL6R, IFI16, and IFIT1B were included (Supplementary Table S1) (Supplementary Figure S1). Multivariate Cox analysis revealed that genes such as PILRA, LILRB2, and BATF were independent prognostic factors for glioma patients (Supplementary Table S2). Additionally, we conducted GSVA analysis to explore the association between CD74 expression and the enrichment of immune processes, which indicated that the higher expression of CD74 indicated a higher enrichment of cytokine production, lymphocyte activation, interferon related signaling pathway, and leukocyte differentiation (Figures 5A–D). These results demonstrated that CD74 was closely associated with immune-related genes and immune processes, indicating the immune characteristics of CD74 in gliomas.

### CD74 expression indicated low tumor purity and high infiltration of immune cells in the glioma microenvironment.

**FIGURE 6:**
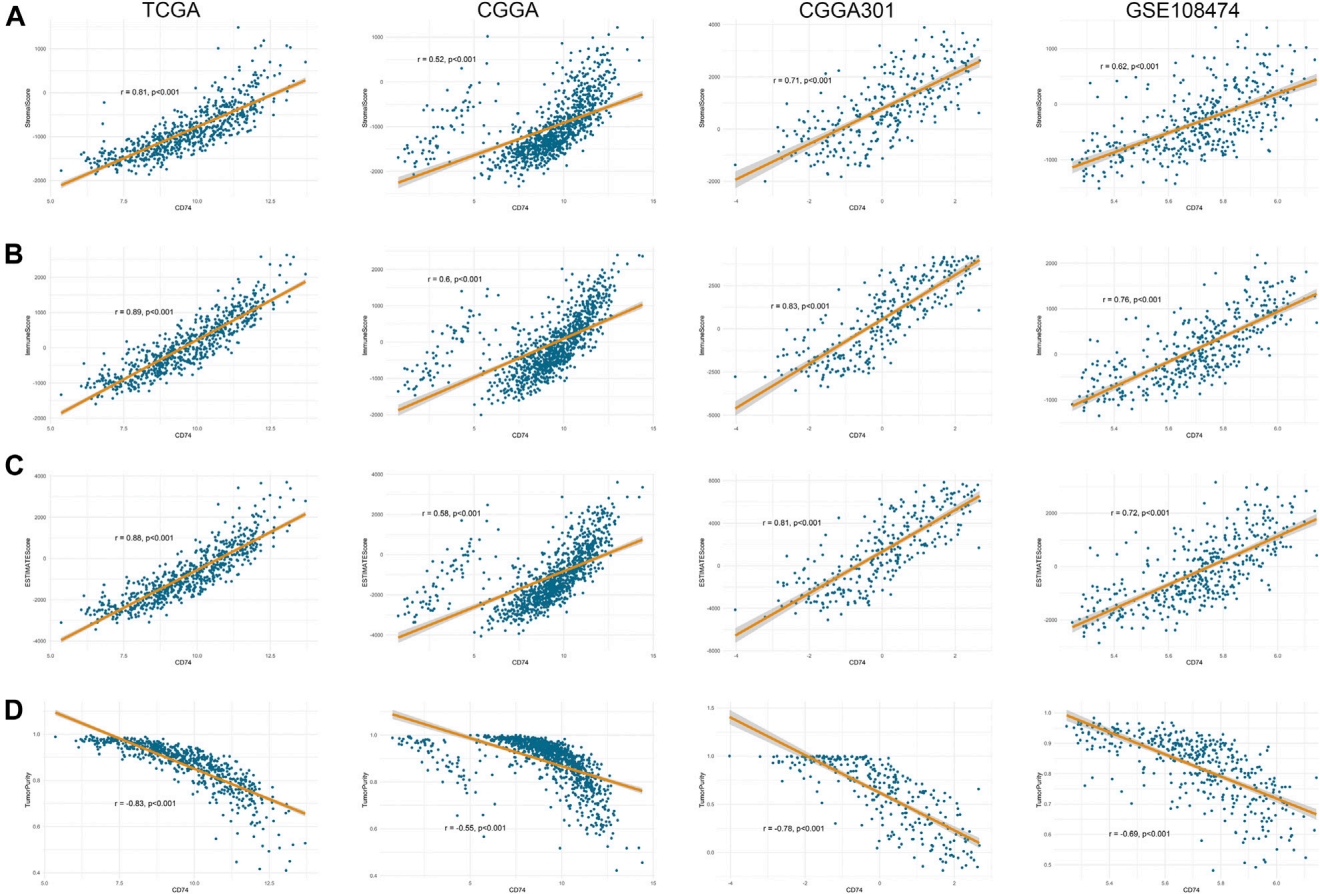
Correlation between CD74 expression and tumor purity. A-D. Tumor purity was assessed by ESTIMATE algorithm. The correlation between CD74 expression and stromal score **(A)**, immune score **(B)**, ESTIMATE score **(C)**, and tumor purity **(D)** in the four datasets.

**FIGURE 7:**
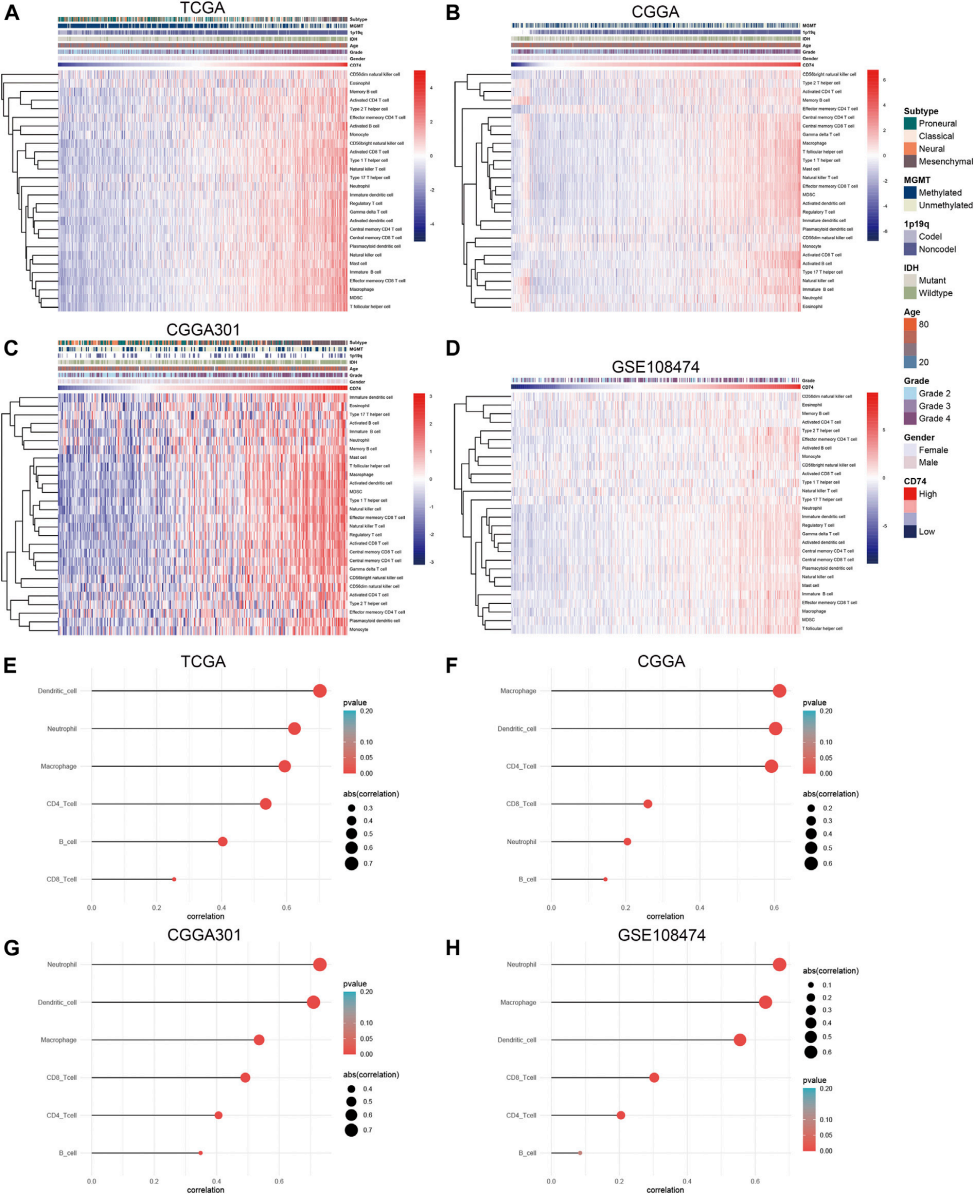
Correlation between CD74 expression and immune infiltration. **(A–D)** The correlation between CD74 expression and immune infiltration estimated by the ssGSEA algorithm. **(E–H)** The correlation between CD74 expression and immune infiltration estimated by the TIMER algorithm.

Further, we employed the ESTIMATE, ssGSEA, and TIMER algorithms to further characterize the immune features of CD74. The stromal and immune scores of each sample were calculated based on their gene expression pattern. The expression of CD74 was significantly positively associated with stromal and immune scores of gliomas (*p* < 0.05) (Figures 6A,B). The ESTIMATE score was calculated as the sum of the stromal and immune scores, which was also positively correlated with the expression of CD74 (*p* < 0.05) (Figure 6C). The tumor purity was calculated based on the ESTIMATE score. CD74 was highly expressed in the low-purity tumor, which indicated a relatively high proportion of stromal and immune cells (*p* < 0.05) (Figure 6D). Besides, the higher expression of CD74 indicated a higher abundance of infiltrated immune cells according to the ssGSEA algorithm (Figures 7A–D). In CD74 high group, the abundance of macrophages, activated dendritic cells, and neutrophils was significantly increased than CD74 low group (*p* < 0.05) (Supplementary Figure S2). In the TIMER algorithm, the abundance of macrophage, dendritic cell, and neutrophil was markedly correlated with CD74 expression (*p* < 0.05) (Figures 7E–H). These findings were further verified by the CIBERSORT algorithm, which indicated a higher abundance of activated dendritic cells, macrophages and neutrophils (*p* < 0.05) (Supplementary Figure S3). These results suggested that CD74 correlated with the infiltration of immune cells such as macrophages, dendritic cells, and neutrophils in the glioma microenvironment.

### Prognostic and Predictive Values of CD74 in Gliomas

**FIGURE 8:**
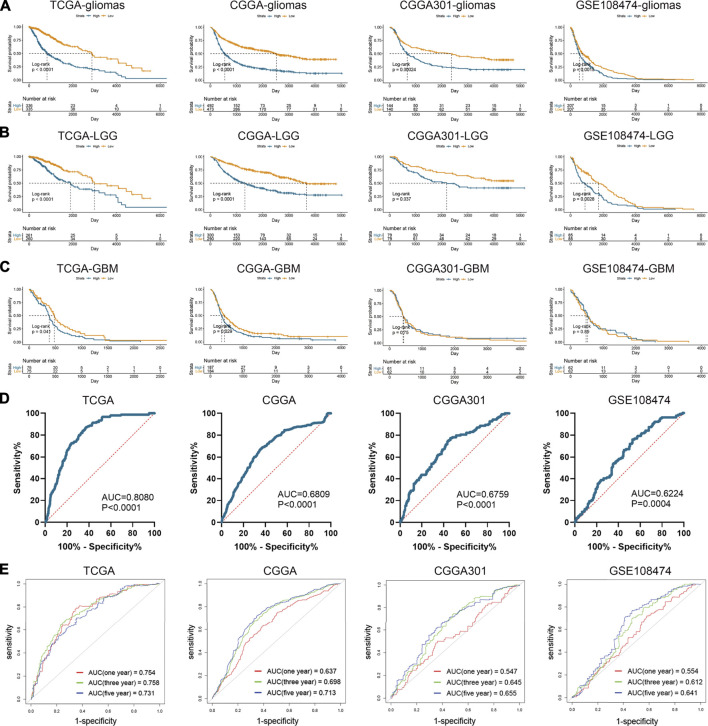
Prognostic and predictive values of CD74 in gliomas. A-C. Kaplan-Meier analysis of CD74 expression in the overall survival of patients with gliomas **(A)**, lower-grade gliomas **(B)**, and glioblastoma **(C)**. D. Patients were divided into glioblastoma and lower-grade glioma groups. ROC analysis was conducted to evaluate the predictive accuracy of CD74 expression for the diagnosis of glioblastoma. E. ROC analysis of CD74 expression in predicting one-, three, and 5-year overall survival of glioma patients. ROC: receiver operating characteristic.

Apart from the immune features, CD74 also exhibited prognostic and predictive values in the OS of glioma patients. Kaplan-Meier analysis revealed that the high expression of CD74 indicated poor prognosis in patients with gliomas and LGG (*p* < 0.05) (Figures 8A,B). While in patients with GBM, this finding was consistent in TCGA and CGGA datasets (*p* < 0.05), and no significant difference was detected in CGGA301 and GSE108474 datasets (*p* > 0.05) (Figure 8C). Multivariate Cox analysis showed that CD74 and tumor grade were independent risk factors for glioma patients in TCGA, CGGA, and GSE108474 datasets (*p* < 0.05) whereas no significant difference was detected in the CGGA301 cohort (*p* > 0.05) (Table 3). Besides, tumor grade was the independent risk factor for glioma patients in the four cohorts (*p* < 0.05). Then we applied CD74 as the potential biomarker to facilitate the diagnosis of GBM, results showed that CD74 had moderate predictive accuracy with the area under the curve (AUC) of 0.808, 0.681, 0.676, and 0.622 in TCGA, CGGA, CGGA301, and GSE108474 datasets, respectively (*p* < 0.05) (Figure 8D). Moreover, CD74 exhibited moderate accuracy in predicting the OS of glioma patients. The AUC of one-, three-, and 5 year survival was 0.754, 0.758, and 0.731, respectively in the TCGA dataset; that in the CGGA cohort was 0.637, 0.698, and 0.713, respectively; that in CGGA301 dataset was 0.547, 0.645, and 0.655, respectively; that in GSE108474 dataset was 0.554, 0.612, and 0.641, respectively (Figure 8E). These results indicated that CD74 could serve as a biomarker to predict the prognosis and facilitate the diagnosis of gliomas.

**TABLE 3:** Multivariate analysis of CD74 and clinical features in four datasets.

Variables	TCGA (*n* = 672)		CGGA (*n* = 1,013)		CGGA301 (*n* = 300)		GSE108474 (*n* = 414)	
	HR (95%CI)	*p* Value	HR (95%CI)	*p* Value	HR (95%CI)	*p* Value	HR (95%CI)	*p* Value
CD74	1.177 (1.045–1.326)	0.007	1.070 (1.019–1.121)	0.018	1.147 (0.896–1.468)	0.277	2.253 (1.219–4.164)	0.010
Age	1.040 (1.028–1.053)	<0.001	1.007 (1–1.015)	0.046	1.025 (0.987–1.064)	0.201	—	—
Grade	1.885 (1.447–2.456)	<0.001	2.149 (1.854–2.491)	<0.001	2.275 (1.473–3.514)	<0.001	1.602 (1.359–1.889)	<0.001
IDH	2.043 (1.277–3.27)	0.003	1.164 (0.927–1.46)	0.191	0.601 (0.245–1.475)	0.266	—	—
1p19q	1.614 (0.944–2.762)	0.080	2.574 (1.845–3.592)	<0.001	4.13 (1.352–12.611)	0.013	—	—
MGMT	1.321 (0.941–1.854)	0.108	1.122 (0.932–1.35)	0.223	2.365 (1.174–4.767)	0.016	—	—

HR: hazard ratio; IDH: isocitrate dehydrogenases; MGMT: O-6-methylguanine-DNA methyltransferase.

## Discussion

Given the fact that glioma patients suffer from poor prognosis and limited therapies, there is a clear urgent need to find novel biomarkers and develop therapeutic strategies. Our study revealed that CD74 was highly expressed in the high-grade glioma and correlated with poor prognosis of glioma patients. Besides, CD74 was positively associated with immune infiltration in the glioma immune microenvironment. These results indicated that CD74 could be used as a potential target for the treatment of glioma and as a novel biomarker to predict prognoses of glioma patients.

Previous studies revealed that CD74 was associated with glioma malignancy and activity of glioma-associated macrophages (Zeiner et al., 2015; Ghoochani et al., 2016; Alban et al., 2020). Our study collected 40 glioma specimens and extracted 2,399 glioma samples from the online databases to explore the expression pattern of CD74 in gliomas. The high expression of CD74 was significantly higher in glioma tissue compared to the normal tissue. CD74 was highly expressed in grade IV, IDH wildtype, 1p19q non-codeletion, and MGMT unmethylated gliomas. Moreover, CD74 was highly expressed in the mesenchymal subtype, which was the most malignant molecular subtype of glioma (Wang et al., 2021). Besides, the protein level of CD74 was markedly elevated in the higher-grade glioma. These results indicated that the high expression of CD74 was associated with a relatively malignant type of glioma. Therefore, CD74 might serve as a biomarker to predict the malignancy of glioma.

As a pivotal component in antigen presentation, CD74 was assumed to have an impact on immune responses and immune infiltrations. A previous study suggested that CD74 was associated with poor prognosis and high tumor-infiltrating leucocyte in breast cancer, which was consistent with our finding (Wang et al., 2017). Our study found that the expression of CD74 was positively associated with the expression of immune checkpoints and inflammatory cytokines. Then, we screened the co-expressed genes of CD74 to further characterize the immune feature of CD74 in gliomas. The GSEA and GSVA analyses showed that the positively co-expressed genes of CD74 were enriched in immune responses and autoimmune diseases. These results preliminarily demonstrated the immune characteristics of CD74 in gliomas. However, it should be noted that these algorithms were conducted based on the expression of pathway-related genes, which could not determine the actual status of these immune processes in different samples. Additional experiments were needed to explore the regulatory effect of CD74 on specific immune processes.

Immune microenvironment was implicated to play an important role in cancer progression (Quail and Joyce, 2017; Zhang et al., 2020a; Zhang et al., 2020b; Wang et al., 2020). CD74 was the receptor of MIF and the CD74-MIF signaling pathway regulated the activity of macrophages and other immune cells (Borghese and Clanchy, 2011; Figueiredo et al., 2018). Therefore, we also explored the correlation between CD74 and the abundance of infiltrated immune cells. The tumor purity of each glioma sample was calculated based on the ESTIMATE algorithm. Since the high immune infiltration indicated low tumor purity, the negative correlation between CD74 and tumor purity suggested that high expression of CD74 was associated with high immune infiltration. With the application of two algorithms, we found that CD74 was markedly positively associated with the infiltration of immune cells such as macrophages, dendritic cells, and neutrophils. Previous studies indicated that macrophages in tumor microenvironment commonly referred to M2 subtype, which exhibited pro-tumoral effects and induced immunosuppressive context (Najafi et al., 2019; Xu et al., 2020b). Moreover, high densities of infiltrating neutrophils were associated with advanced stages of cancers including glioma (Shaul and Fridlender, 2019). Additionally, Zhang et al. (2017) revealed that tumor purity was lowest in grade IV glioma and highest in grade II glioma, and the low tumor purity indicated a high enrichment of macrophages, activated dendritic cells, and neutrophils (Zhang et al., 2017). Therefore, the infiltration of macrophages, dendritic cells, and neutrophils indicated high malignancy of glioma, which was consistent with our findings that CD74 was positively associated with the malignancy of glioma and the infiltration of macrophages, dendritic cells, and neutrophils. However, it should be noted that the infiltration of immune cells was assessed using different algorithms. These algorithms were conducted based on the expression of genes that were associated with the activity of related immune cells. Therefore, it was hard to determine the exact population of immune cells in different samples. However, through this large-scale analysis, the immune characteristics of CD74 were preliminarily demonstrated, which could provide a reference for further investigation of the regulatory association between CD74 and immune cells.

The low tumor purity has been implicated to be associated with poor prognosis in glioma patients (Zhang et al., 2017; Xu et al., 2021; Zhang et al., 2021; Kang et al., 2021). Since high expression of CD74 indicated low tumor purity, we further explored the clinical significance of CD74 in glioma patients. In glioma and LGG patients, CD74 was significantly associated with survival rates. However, the finding was diverse in GBM patients. This might be due to the intense malignancy of GBM and the single biomarker had a limited ability to stratify the survival of GBM patients. Moreover, multivariate analyses indicated that CD74 was an independent risk factor for glioma patients in the TCGA, CGGA, and GSE108474 datasets, but not in the CGGA301 dataset. Since we included the status of IDH, 1p19q, and MGMT in multivariate analyses, the number of samples that had complete information in the CGGA301 dataset was relatively limited as shown in Table 1, which might lead to the discrepancy of the results in the CGGA301 dataset compared with other datasets. Additionally, CD74 exhibited moderate predictive accuracy in the diagnosis of GBM and the prognosis of glioma patients. Compared with integrated signature, the predictive accuracy of CD74 was relatively low. Cheng et al. constructed a risk signature based on the expression of eight immune-related genes, which could predict the prognosis and the efficacy of chemoradiotherapy in GBM patients (Cheng et al., 2016). Similarly, Zuo et al. (2019) reported a six-gene signature that could predict the OS and therapeutic responses of GBM patients (Zuo et al., 2019). The AUC value of their constructed signatures in predicting long-term survival was higher than that of our study. Therefore, the predictive value of a single biomarker was limited. However, CD74 still had some merits in predicting the diagnosis and prognosis of gliomas and could be used as a potential target for glioma treatment.

However, additional experiments would help provide a better understanding of the underlying mechanism of CD74 in the development and progression of gliomas. Although CD74 was proven to be associated with several immune processes, the effect of CD74 on specific immune cells required further investigation.

## Conclusion

To sum up, we conducted a large-scale analysis to characterize the immune and clinical features of CD74 in gliomas. The high expression of CD74 was associated with poor prognosis and high immune infiltration. CD74 could be used as a potential target for glioma treatment and as a biomarker to predict the prognosis of glioma patients.

## Data Availability

Publicly available datasets were analyzed in this study. The data are accessible in TCGA and CGGA databases.

## Abbreviations

CD74, cluster of differentiation 74; IDH, isocitrate dehydrogenase; MGMT, O6-methylguanine-DNA methyltransferase; HLA, human lymphocyte antigen; MIF, macrophage migration inhibitory factor; TCGA, The Cancer Genome Atlas; CGGA, Chinese Glioma Genome Atlas; GEO, Gene-Expression Omnibus; GTEx, Genotype-Tissue Expression; TPM, transcripts per kilobase million; IHC, immunohistochemistry; DAB, diaminobenzidine tetrahydrochloride; GSVA, gene set variation analysis; GSEA, gene sets enrichment analysis; GO, Gene Ontology; KEGG, Kyoto Encyclopedia of Genes and Genomes; FDR, false discovery rate; ESTIMATE, Estimation of STromal and Immune cells in MAlignant Tumor tissues using Expression data; ssGSEA, single-sample gene-set enrichment analysis; TIMER, Tumor IMmune Estimation Resource; OS, overall survival; ROC, receiver operating characteristic, LGG, lower-grade glioma; TIM-3, T cell immunoglobulin domain and mucin domain-3; AUC, area under the curve.

## References

[B1] Alban T. J. Bayik D. Otvos B. Rabljenovic A. Leng L. Jia-Shiun L. (2020). Glioblastoma Myeloid-Derived Suppressor Cell Subsets Express Differential Macrophage Migration Inhibitory Factor Receptor Profiles that Can Be Targeted to Reduce Immune Suppression. Front. Immunol. 11, 1191. 10.3389/fimmu.2020.01191 32625208 PMC7315581

[B2] Barbie D. A. Tamayo P. Boehm J. S. Kim S. Y. Moody S. E. Dunn I. F. (2009). Systematic RNA Interference Reveals that Oncogenic KRAS-Driven Cancers Require TBK1. Nature 462 (7269), 108–112. 10.1038/nature08460 19847166 PMC2783335

[B3] Binsky I. Haran M. Starlets D. Gore Y. Lantner F. Harpaz N. (2007). IL-8 Secreted in a Macrophage Migration-Inhibitory Factor- and CD74-dependent Manner Regulates B Cell Chronic Lymphocytic Leukemia Survival. Proc. Natl. Acad. Sci. 104 (33), 13408–13413. 10.1073/pnas.0701553104 17686984 PMC1948950

[B4] Blackburn S. D. Shin H. Haining W. N. Zou T. Workman C. J. Polley A. (2009). Coregulation of CD8+ T Cell Exhaustion by Multiple Inhibitory Receptors during Chronic Viral Infection. Nat. Immunol. 10 (1), 29–37. 10.1038/ni.1679 19043418 PMC2605166

[B5] Bleeker F. E. Molenaar R. J. Leenstra S. (2012). Recent Advances in the Molecular Understanding of Glioblastoma. J. Neurooncol. 108 (1), 11–27. 10.1007/s11060-011-0793-0 22270850 PMC3337398

[B6] Borghese F. Clanchy F. I. (2011). CD74: an Emerging Opportunity as a Therapeutic Target in Cancer and Autoimmune Disease. Expert Opin. Ther. Targets 15 (3), 237–251. 10.1517/14728222.2011.550879 21208136

[B7] Chen H. Thomas C. Munoz F. A. Alexandrescu S. Horbinski C. M. Olar A. (2019). Polysomy Is Associated with Poor Outcome in 1p/19q Codeleted Oligodendroglial Tumors. Neuro Oncol. 21 (9), 1164–1174. 10.1093/neuonc/noz098 31140557 PMC7571489

[B8] Cheng W. Ren X. Zhang C. Cai J. Liu Y. Han S. (2016). Bioinformatic Profiling Identifies an Immune-Related Risk Signature for Glioblastoma. Neurology 86 (24), 2226–2234. 10.1212/wnl.0000000000002770 27225222

[B9] Cresswell P. (1994). Assembly, Transport, and Function of MHC Class II Molecules. Annu. Rev. Immunol. 12, 259–291. 10.1146/annurev.iy.12.040194.001355 8011283

[B10] Farr L. Ghosh S. Moonah S. (2020). Role of MIF Cytokine/CD74 Receptor Pathway in Protecting against Injury and Promoting Repair. Front. Immunol. 11, 1273. 10.3389/fimmu.2020.01273 32655566 PMC7325688

[B11] Figueiredo C. R. Azevedo R. A. Mousdell S. Resende-Lara P. T. Ireland L. Santos A. (2018). Blockade of MIF-CD74 Signalling on Macrophages and Dendritic Cells Restores the Antitumour Immune Response against Metastatic Melanoma. Front. Immunol. 9, 1132. 10.3389/fimmu.2018.01132 29875777 PMC5974174

[B12] Ghoochani A. Schwarz M. A. Yakubov E. Engelhorn T. Doerfler A. Buchfelder M. (2016). MIF-CD74 Signaling Impedes Microglial M1 Polarization and Facilitates Brain Tumorigenesis. Oncogene 35 (48), 6246–6261. 10.1038/onc.2016.160 27157615

[B13] Goodenberger M. L. Jenkins R. B. (2012). Genetics of Adult Glioma. Cancer Genet. 205 (12), 613–621. 10.1016/j.cancergen.2012.10.009 23238284

[B14] Hänzelmann S. Castelo R. Guinney J. (2013). GSVA: Gene Set Variation Analysis for Microarray and RNA-Seq Data. BMC Bioinformatics 14, 7. 10.1186/1471-2105-14-7 23323831 PMC3618321

[B15] Hegi M. E. Diserens A.-C. Gorlia T. Hamou M.-F. de Tribolet N. Weller M. (2005). MGMTGene Silencing and Benefit from Temozolomide in Glioblastoma. N. Engl. J. Med. 352 (10), 997–1003. 10.1056/nejmoa043331 15758010

[B16] Kang Y. Huang J. Liu Y. Zhang N. Cheng Q. Zhang Y. (2021). Integrated Analysis of Immune Infiltration Features for Cervical Carcinoma and Their Associated Immunotherapeutic Responses. Front. Cel Dev. Biol. 9, 573497. 10.3389/fcell.2021.573497 PMC8063060 33898414

[B17] Li T. Fu J. Zeng Z. Cohen D. Li J. Chen Q. (2020). TIMER2.0 for Analysis of Tumor-Infiltrating Immune Cells. Nucleic Acids Res. 48 (W1), W509–W514. 10.1093/nar/gkaa407 32442275 PMC7319575

[B18] Louis D. N. Perry A. Reifenberger G. von Deimling A. Figarella-Branger D. Cavenee W. K. (2016). The 2016 World Health Organization Classification of Tumors of the Central Nervous System: a Summary. Acta Neuropathol. 131 (6), 803–820. 10.1007/s00401-016-1545-1 27157931

[B19] Margolin A. A. Nemenman I. Basso K. Wiggins C. Stolovitzky G. Dalla Favera R. (2006). ARACNE: an Algorithm for the Reconstruction of Gene Regulatory Networks in a Mammalian Cellular Context. BMC Bioinformatics 7 Suppl 1 (Suppl. 1), S7. 10.1186/1471-2105-7-S1-S7 PMC1810318 16723010

[B20] Meyer-Siegler K. L. Iczkowski K. A. Leng L. Bucala R. Vera P. L. (2006). Inhibition of Macrophage Migration Inhibitory Factor or its Receptor (CD74) Attenuates Growth and Invasion of DU-145 Prostate Cancer Cells. J. Immunol. 177 (12), 8730–8739. 10.4049/jimmunol.177.12.8730 17142775

[B21] Molenaar R. J. Radivoyevitch T. Maciejewski J. P. van Noorden C. J. F. Bleeker F. E. (2014). The Driver and Passenger Effects of Isocitrate Dehydrogenase 1 and 2 Mutations in Oncogenesis and Survival Prolongation. Biochim. Biophys. Acta (Bba) - Rev. Cancer 1846 (2), 326–341. 10.1016/j.bbcan.2014.05.004 24880135

[B22] Molenaar R. J. Verbaan D. Lamba S. Zanon C. Jeuken J. W. M. Boots-Sprenger S. H. E. (2014). The Combination of IDH1 Mutations and MGMT Methylation Status Predicts Survival in Glioblastoma Better Than Either IDH1 or MGMT Alone. Neuro Oncol. 16 (9), 1263–1273. 10.1093/neuonc/nou005 24510240 PMC4136888

[B23] Nagata S. Jin Y.-F. Yoshizato K. Tomoeda M. Song M. Iizuka N. (2009). CD74 Is a Novel Prognostic Factor for Patients with Pancreatic Cancer Receiving Multimodal Therapy. Ann. Surg. Oncol. 16 (9), 2531–2538. 10.1245/s10434-009-0532-3 19499276

[B24] Najafi M. Hashemi Goradel N. Farhood B. Salehi E. Nashtaei M. S. Khanlarkhani N. (2019). Macrophage Polarity in Cancer: A Review. J. Cel Biochem 120 (3), 2756–2765. 10.1002/jcb.27646 30270458

[B25] Newman A. M. Liu C. L. Green M. R. Gentles A. J. Feng W. Xu Y. (2015). Robust Enumeration of Cell Subsets from Tissue Expression Profiles. Nat. Methods 12 (5), 453–457. 10.1038/nmeth.3337 25822800 PMC4739640

[B26] Ohgaki H. Kleihues P. (2005). Population-based Studies on Incidence, Survival Rates, and Genetic Alterations in Astrocytic and Oligodendroglial Gliomas. J. Neuropathol. Exp. Neurol. 64 (6), 479–489. 10.1093/jnen/64.6.479 15977639

[B27] Quail D. F. Joyce J. A. (2017). The Microenvironmental Landscape of Brain Tumors. Cancer Cell 31 (3), 326–341. 10.1016/j.ccell.2017.02.009 28292436 PMC5424263

[B28] Shaul M. E. Fridlender Z. G. (2019). Tumour-associated Neutrophils in Patients with Cancer. Nat. Rev. Clin. Oncol. 16 (10), 601–620. 10.1038/s41571-019-0222-4 31160735

[B29] Smoll N. R. Gautschi O. P. Schatlo B. Schaller K. Weber D. C. (2012). Relative Survival of Patients with Supratentorial Low-Grade Gliomas. Neuro-Oncology 14 (8), 1062–1069. 10.1093/neuonc/nos144 22773277 PMC3408266

[B30] Tanese K. Hashimoto Y. Berkova Z. Wang Y. Samaniego F. Lee J. E. (2015). Cell Surface CD74-MIF Interactions Drive Melanoma Survival in Response to Interferon-γ. J. Invest. Dermatol. 135 (11), 2775–2784. 10.1038/jid.2015.204 26039541 PMC4640965

[B31] Tang Z. Li C. Kang B. Gao G. Li C. Zhang Z. (2017). GEPIA: a Web Server for Cancer and normal Gene Expression Profiling and Interactive Analyses. Nucleic Acids Res. 45 (W1), W98–W102. 10.1093/nar/gkx247 28407145 PMC5570223

[B32] Thakkar J. P. Dolecek T. A. Horbinski C. Ostrom Q. T. Lightner D. D. Barnholtz-Sloan J. S. (2014). Epidemiologic and Molecular Prognostic Review of Glioblastoma. Cancer Epidemiol. Biomarkers Prev. 23 (10), 1985–1996. 10.1158/1055-9965.epi-14-0275 25053711 PMC4185005

[B33] Verhaak R. G. W. Hoadley K. A. Purdom E. Wang V. Qi Y. Wilkerson M. D. (2010). Integrated Genomic Analysis Identifies Clinically Relevant Subtypes of Glioblastoma Characterized by Abnormalities in PDGFRA, IDH1, EGFR, and NF1. Cancer Cell 17 (1), 98–110. 10.1016/j.ccr.2009.12.020 20129251 PMC2818769

[B34] Wang K. Y. Huang R. Y. Tong X. Z. Zhang K. N. Liu Y. W. Zeng F. (2019). Molecular and Clinical Characterization of TMEM71 Expression at the Transcriptional Level in Glioma. CNS Neurosci. Ther. 25 (9), 965–975. 10.1111/cns.13137 31180187 PMC6698980

[B35] Wang Y. Wang Y. Xu C. Liu Y. Huang Z. (2020). Identification of Novel Tumor-Microenvironment-Regulating Factor that Facilitates Tumor Immune Infiltration in Colon Cancer. Mol. Ther. - Nucleic Acids 22, 236–250. 10.1016/j.omtn.2020.08.029 33230430 PMC7515980

[B36] Wang Z.-Q. Milne K. Webb J. R. Watson P. H. (2017). CD74 and Intratumoral Immune Response in Breast Cancer. Oncotarget 8 (8), 12664–12674. 10.18632/oncotarget.8610 27058619 PMC5355043

[B37] Wang Z. Zhang H. Xu S. Liu Z. Cheng Q. (2021). The Adaptive Transition of Glioblastoma Stem Cells and its Implications on Treatments. Sig Transduct Target. Ther. 6 (1), 124. 10.1038/s41392-021-00491-w PMC7985200 33753720

[B38] Xu S. Tang L. Dai G. Luo C. Liu Z. (2020). Expression of m6A Regulators Correlated with Immune Microenvironment Predicts Therapeutic Efficacy and Prognosis in Gliomas. Front. Cel Dev. Biol. 8, 594112. 10.3389/fcell.2020.594112 PMC7683617 33240891

[B39] Xu S. Tang L. Dai G. Luo C. Liu Z. (2021). Immune-related Genes with APA in Microenvironment Indicate Risk Stratification and Clinical Prognosis in Grade II/III Gliomas. Mol. Ther. - Nucleic Acids 23, 1229–1242. 10.1016/j.omtn.2021.01.033 33665000 PMC7900014

[B40] Xu S. Tang L. Li X. Fan F. Liu Z. (2020). Immunotherapy for Glioma: Current Management and Future Application. Cancer Lett. 476, 1–12. 10.1016/j.canlet.2020.02.002 32044356

[B41] Yoshihara K. Shahmoradgoli M. Martínez E. Vegesna R. Kim H. Torres-Garcia W. (2013). Inferring Tumour Purity and Stromal and Immune Cell Admixture from Expression Data. Nat. Commun. 4, 2612. 10.1038/ncomms3612 24113773 PMC3826632

[B42] Zeiner P. S. Preusse C. Blank A.-E. Zachskorn C. Baumgarten P. Caspary L. (2015). MIF Receptor CD74 Is Restricted to Microglia/Macrophages, Associated with a M1-Polarized Immune Milieu and Prolonged Patient Survival in Gliomas. Brain Pathol. 25 (4), 491–504. 10.1111/bpa.12194 25175718 PMC8029437

[B43] Zhang C. Cheng W. Ren X. Wang Z. Liu X. Li G. (2017). Tumor Purity as an Underlying Key Factor in Glioma. Clin. Cancer Res. 23 (20), 6279–6291. 10.1158/1078-0432.ccr-16-2598 28754819

[B44] Zhang N. Dai Z. Wu W. Wang Z. Cao H. Zhang Y. (2021). The Predictive Value of Monocytes in Immune Microenvironment and Prognosis of Glioma Patients Based on Machine Learning. Front. Immunol. 12, 656541. 10.3389/fimmu.2021.656541 33959130 PMC8095378

[B45] Zhang X. Shi M. Chen T. Zhang B. (2020). Characterization of the Immune Cell Infiltration Landscape in Head and Neck Squamous Cell Carcinoma to Aid Immunotherapy. Mol. Ther. - Nucleic Acids 22, 298–309. 10.1016/j.omtn.2020.08.030 33230435 PMC7522342

[B46] Zhang Y. Yang M. Ng D. M. Haleem M. Yi T. Hu S. (2020). Multi-omics Data Analyses Construct TME and Identify the Immune-Related Prognosis Signatures in Human LUAD. Mol. Ther. - Nucleic Acids 21, 860–873. 10.1016/j.omtn.2020.07.024 32805489 PMC7452010

[B47] Zuo S. Zhang X. Wang L. (2019). A RNA Sequencing-Based Six-Gene Signature for Survival Prediction in Patients with Glioblastoma. Sci. Rep. 9 (1), 2615. 10.1038/s41598-019-39273-4 30796273 PMC6385312

