# Supplementary figures and images for: CD74 Correlated With Malignancies and Immune Microenvironment in Gliomas
Source: Front Mol Biosci. 2021 Sep 1;8:706949. doi: 10.3389/fmolb.2021.706949 (PMC8440887; doi:10.3389/fmolb.2021.706949)

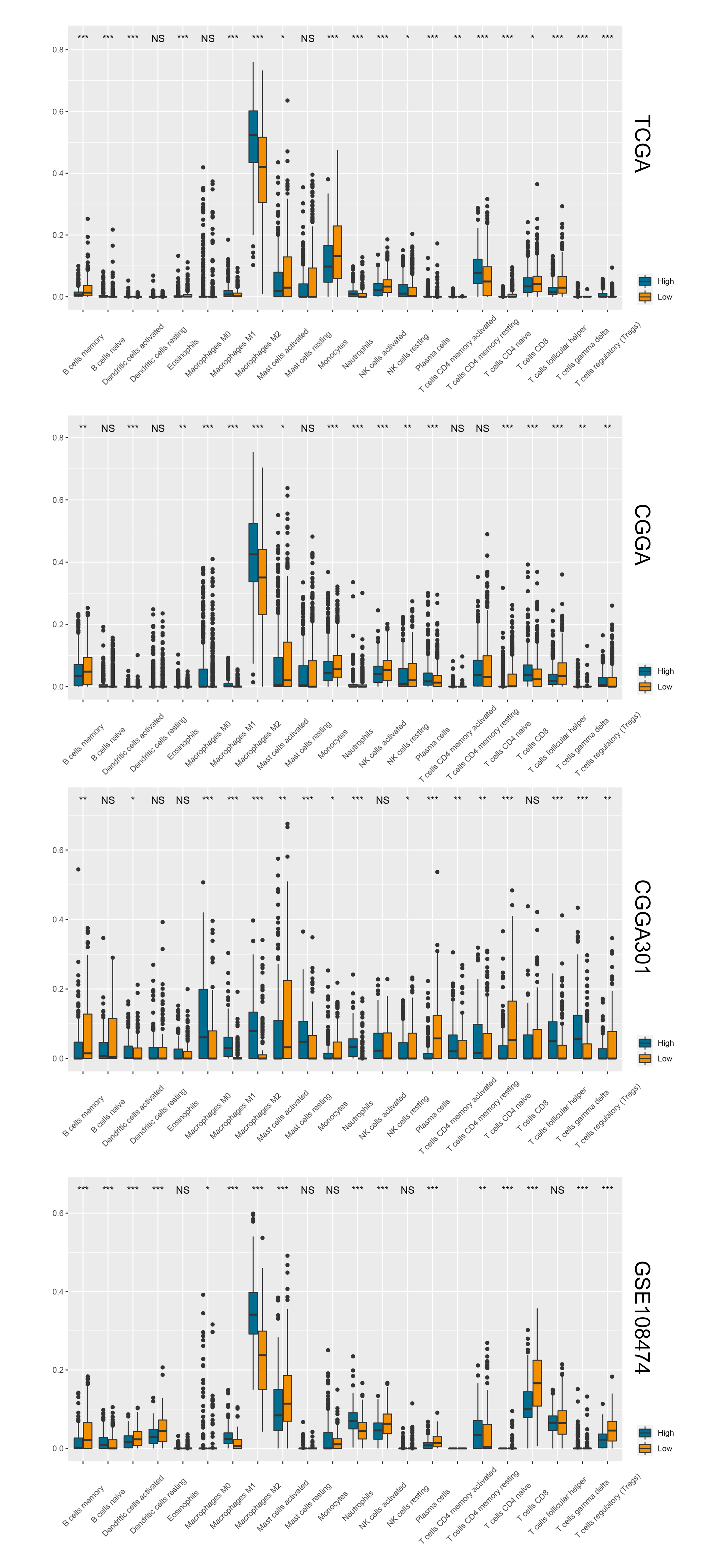

Supplement: Supplementary file 2 [file Image3.TIF]

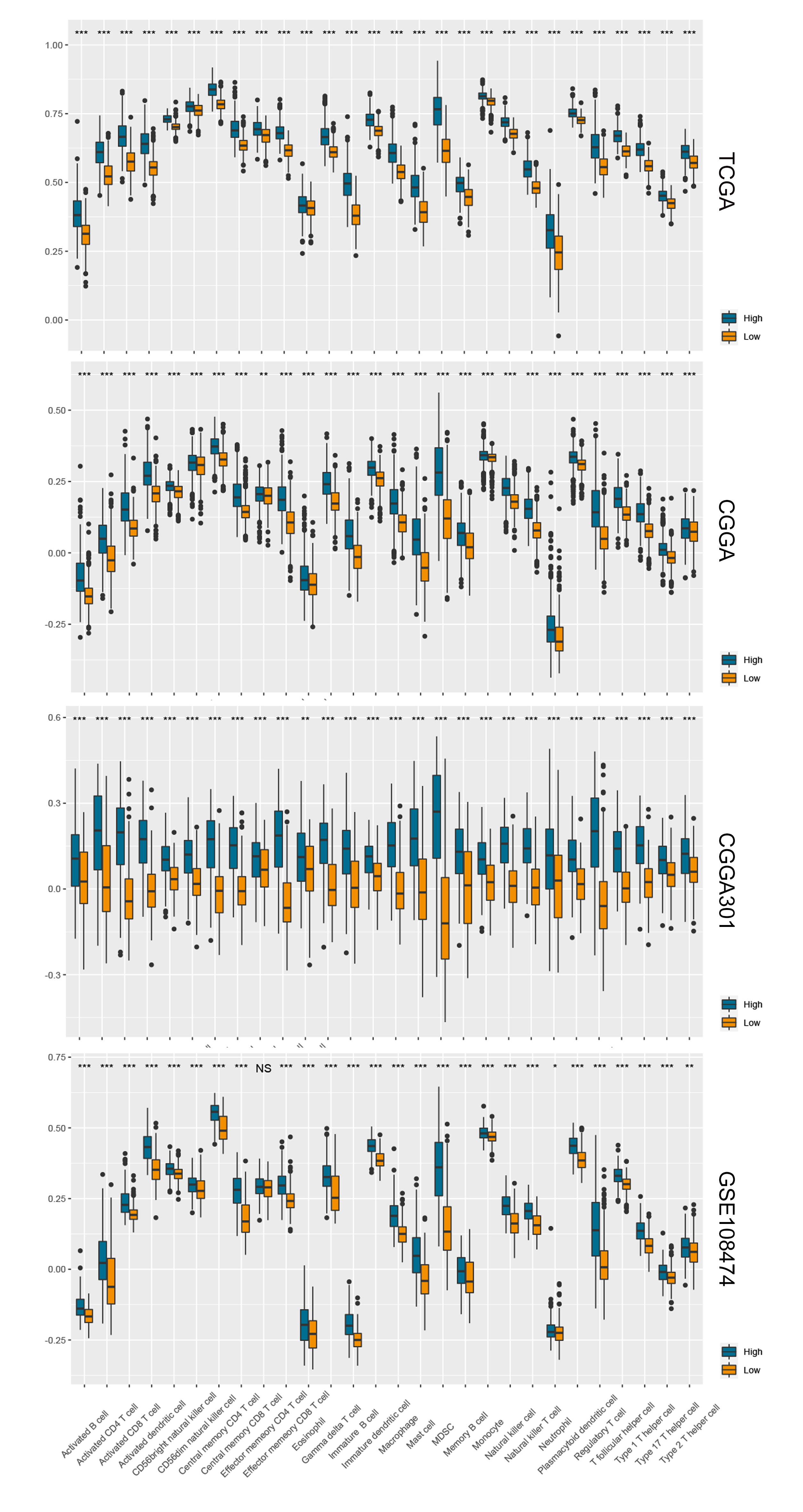

Supplement: Supplementary file 3 [file Image2.TIF]

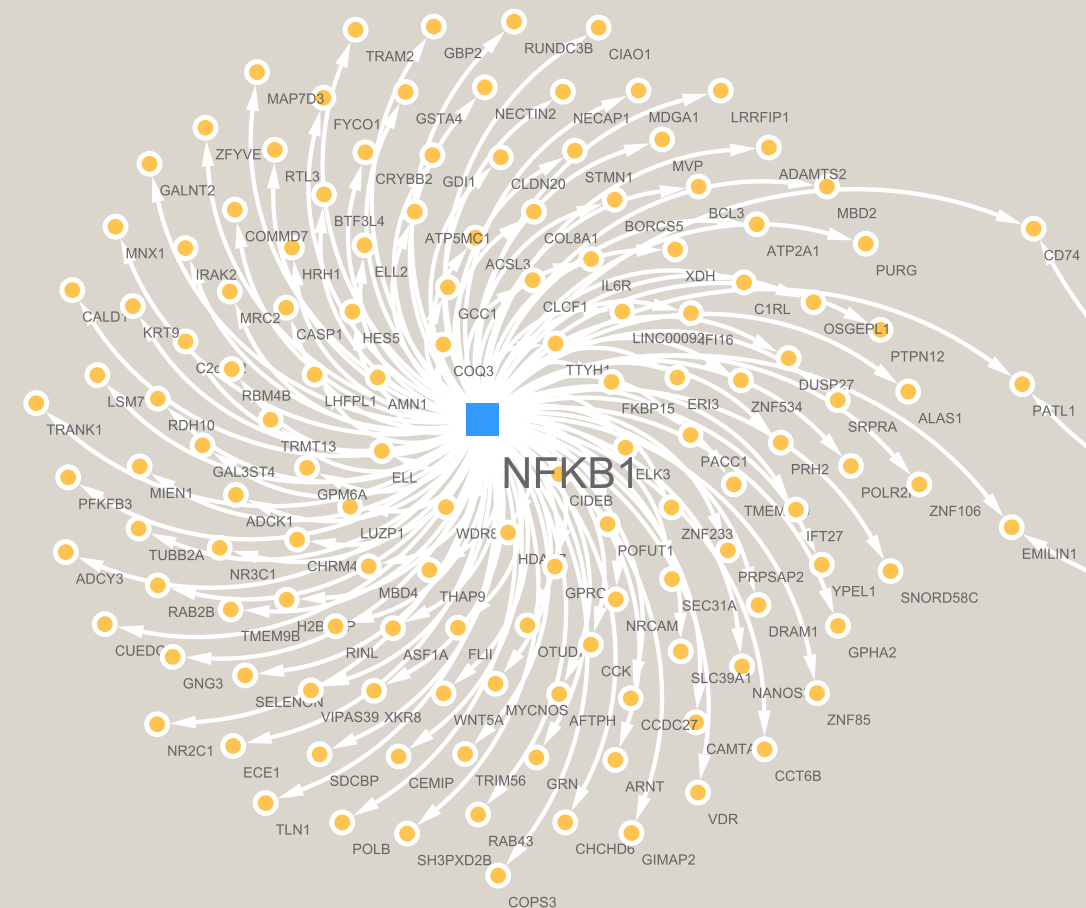

Supplement: Supplementary file 5 [file Image1.PDF]
